# Screen Time Is Associated with Altered Neural Maturation in a Massive Adolescent Cohort

**DOI:** 10.3390/children13070969

**Published:** 2026-07-22

**Authors:** Alexander S. Atalay, Benjamin T. Newman, T. Jason Druzgal

**Affiliations:** Department of Radiology and Medical Imaging, University of Virginia, Charlottesville, VA 22903, USAtjd4m@virginia.edu (T.J.D.)

**Keywords:** screen time, adolescence, neuroimaging, ABCD Study, cognitive neuroscience, diffusion MRI

## Abstract

**Highlights:**

**What are the main findings?**

- Screen time use by adolescents was associated with accelerated developmental trajectories of cellular microstructure in reward-system-associated cortical regions and decelerated developmental trajectories in cognitive control-system-associated cortical regions.
- Advanced diffusion cellular microstructure techniques were more sensitive to this relationship compared to gray matter density or traditional diffusion tensor imaging.

**What is the implication of the main findings?**

- Increased screen time use by adolescents may alter typical age-related cortical developmental trajectories in brain regions functionally associated with reward and cognitive control.

**Abstract:**

Background: A growing body of literature associates increases in electronic screen time with a vast array of psychological consequences amongst adolescents, but little is known about the neurological underpinnings of this relationship. Methods: This longitudinal study examines structural and diffusion brain MRI scans from two timepoints collected from the Adolescent Brain Cognitive Development (ABCD) Study—a large, multi-site study with thousands of participants. By assessing both gray matter density (GMD) and gray matter measurements of diffusion microstructure in the adolescent brain, we describe how the developmental trajectory of the brain changes with screen-based media consumption at the subcellular level. Gray matter microstructure was measured across 13 bilateral regions functionally implicated with screen time use and associated with either the control or reward system. Results: After controlling for age, sex, total brain volume, scanning site, sibling relationships, physical activity, and socioeconomic status, this study finds significant positive correlations between increased screen time and axonal signal across six of the 13 regions selected, while also finding significantly decreased intracellular signaling in eight regions, primarily in regions functionally associated with cognitive control. Comparing these associations to normal developmental trajectories suggests adolescent age-related brain development may be accelerated by increased screen time in brain areas associated with reward processing, while age-related brain development may be decelerated in regions of the control system. Highlighting the sensitivity of microstructural analysis, no significant relationships with increased screen time were found using GMD or fractional anisotropy. Conclusions: This work suggests that increased screen usage during adolescent development has a complex association with brain tissue that cannot be completely described by traditional quantifications of tissue microstructure.

## 1. Introduction

Adolescence is a crucial period for the growth and maturation of the brain and the development of cognitive capabilities that affect the remainder of the lifespan. As a result of technological advances and the COVID-19 pandemic, screen time usage is at an all-time high [1]. In adolescents, increased screen time has been linked to lower psychological well-being [2], but little is known about the relationship between increased screen time and neural development, and previous neuroimaging studies on the subject have focused on either small or cross-sectional subject pools [3]. This Study examines structural and diffusion MRI scans from the longitudinal Adolescent Brain Cognitive Development (ABCD) study cohort to describe the effects of screen time on both gray matter density (GMD) and diffusion MRI (dMRI) tissue microstructure in the adolescent brain. A total of 13 regions of interest (ROIs) identified by previous studies were selected for investigation based on either their functional involvement during screen time usage or structural associations with isolated screen-oriented activities in adolescence [3]. These ROIs were classified based on primary involvement in either the cognitive control system or reward processing system—two networks that have demonstrated substantial relationships with screen-based activates in adolescents across multiple studies [3].

### 1.1. The Effects of Screen Time on Adolescent Development

The adolescent brain undergoes substantial neuroanatomical changes at both global and cellular scales during development. Developing brains typically present increases in total brain volume accompanied by non-linear global increases in white matter (WM) volume and decreases in gray matter (GM) volume [4,5,6]. While these relationships are typically spatially and temporally dependent on region and age, they offer insight into the functional reorganization of the brain during development, and non-traditional development trajectories can predict a variety of neuropsychiatric pathologies such as schizophrenia and bipolar disorder [7]. Accompanying the growth of cognitive abilities, the cognitive control system, responsible for the ability to select behaviors based on thoughts, emotions, and social context [8], matures substantially during adolescence. Furthermore, the maturation of affective control—the application of cognitive control to emotional contexts—drives developmental changes in emotional regulation tendencies during adolescence, specifically relying on changes in connectivity between prefrontal regions and regions implicated in emotion and reward processing [3,9].

Increased screen time has been associated with a host of negative psychological and physiological outcomes in adolescents, including poor sleep, high blood pressure, obesity, poor stress regulation, and insulin resistance [7]. Symptoms of attention deficit/hyperactivity disorder (ADHD), a neurodevelopmental disorder characterized by inattention, hyperactivity, and impulsivity, have also been linked to screen media consumption [10]. Relationships have been found between overall screen time and increased symptoms of depression and suicidal behavior among adolescents [11]. While these studies demonstrate a clear association between screen time and adverse health outcomes, the mechanism by which screen time influences brain development is largely undescribed. Despite the growing concern surrounding general screen use in adolescents and the mounting evidence for its consequences, little is known about the neurological basis for screen-driven changes during development.

Neuroimaging research focused on structural changes associated with screen time has been heterogeneous, with significant associations primarily being found when subjects are followed longitudinally. In a cross-sectional analysis of frequency of internet use and development of brain structures in adolescents, there were no significant associations between a higher frequency of internet use and regional gray matter or white matter volumes calculated through voxel-based morphometry (VBM). However, longitudinally, a higher frequency of internet usage predicted change in regional gray matter and white matter volumes in a widespread anatomical cluster, including the orbitofrontal cortex (OFC), temporoparietal junction (TPJ), insula, and amygdala [12]. Cross-sectional research focused on the effects of screen time on white matter microstructure quantified through fractional anisotropy (FA) found no significant relationships [13]. Previous research has also evaluated the relationship between screen time and functional connectivity. In a 2018 study involving 19 10-year-old children, screen time was related to lower connectivity between the left visual word form area and the anterior cingulate cortex (ACC), inferior frontal gyrus (IFG), and insula [14]. Research investigating the effects of media-related tasks on the adolescent brain using a task-based fMRI approach have established robust relationships between social media use and reward network recruitment [15,16], as well as an association between sedentary screen time and decreased impulse control [17]. In research comparing adolescent subjects with internet addictions to healthy controls, relationships were found between problematic internet usage and lower gray matter density in the left ACC, posterior cingulate cortex, and insula [18], as well as a reduction in cortical thickness in the OFC [19]. Finally, in a network-scale analysis, frequency and duration of screen-based media consumption was correlated with lower overall efficiency of the cognitive control system [3].

**Figure 1:**
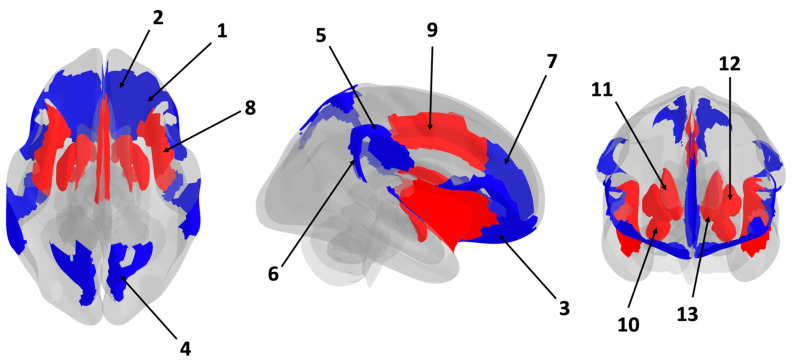
ROI map overlay showing the spatial relationships between selected regions. ROIs are color-filled based on associations with control (blue) or reward (red) systems. Number labels are listed in Table 1.

Existing literature on the associations between screen time and adolescent brain development were recently summarized through a scoping review of 16 neuroimaging studies, in which 13 bilateral ROIs were identified as demonstrating longitudinal relationships with screen time [3]. Of the 13 regions of interest identified, seven were labeled as being involved in the cognitive control system, and six in the reward system. The control system regions identified and further investigated in this study include the ventromedial prefrontal cortex (vmPFC), orbitofrontal cortex (OFC), inferior frontal gyrus (IFG), superior parietal lobule (SPL), inferior parietal lobe (IPL), temporoparietal junction (TPJ), and the anterior cingulate cortex (ACC). The reward system ROIs are the insula, dorsal ACC (dACC), amygdala, caudate nucleus (CAU), putamen, and nucleus accumbens (NAc). A summary of the ROIs, their functional associations, and their neuroanatomical arrangement, can be found in Figure 1 and Table 1.

### 1.2. Microstructural Analysis

Modern MRI analysis pipelines allow for quantification of detailed neuroanatomical characteristics through structural imaging. Two such techniques are voxel-based morphometry [20] (VBM), which allows for the calculation of cortical or subcortical gray matter density (GMD) from structural MRI, and three-tissue constrained spherical deconvolution (3T-CSD) [21,22], which can quantify subcellular components of brain microstructure from dMRI. GMD measures the signal strength in gray matter regions of the brain, acting as a quantification for the general tissue environment. Even greater insight is provided by 3T-CSD, which estimates the proportion of three cellular microenvironments found in the brain: intracellular isotropic (ICI), intracellular anisotropic (ICA), and extracellular isotropic (ECI). These metrics represent signal fractions of water inside cell bodies and structures, water inside axons, and freely diffusing extracellular water, respectively. By tracking the changes in these tissue environments, we can gain insight into what is happening in the brain at a subcellular level.

Based on previous results and established typical developmental trajectories, we hypothesize that subjects with increased screen time will demonstrate reduced or delayed neural development longitudinally. More specifically, we expect to observe a significantly smaller decrease in GMD and ICI signal fraction as well as a significantly smaller increase in ICA signal fraction than observed during typical development.

## 2. Methods

### 2.1. Participants

This Study utilized data obtained from the Adolescent Brain Cognitive Development (ABCD) study [23], a publicly available, longitudinal, neuroimaging and demographic study examining child development and factors leading to substance abuse. As all subjects are de-identified, separate use of the ABCD Study data does not require IRB approval. Due to storage and computational limitations and to avoid manufacturer and sequence differences, including field gradient strength, TE, and TR that have previously been demonstrated to affect the outcomes of three-tissue constrained spherical deconvolution model (3T-CSD) signal fraction results, only subjects from the most common scanner type, Siemens, proceeded to analysis [24,25]. Additional detail on subject inclusion and exclusion during image processing on the baseline sample has previously been published [25]. Subjects missing any relevant data field or additional imaging timepoint were removed from the analysis. Though the inclusion of Siemens scanners only may limit the external generalizability, this is not likely more restrictive than examining subject cohorts with greatly differing b-value shells or other variable imaging parameters known to affect diffusion signal relationships between intracellular and extracellular signal compartments [26,27,28].

Following a semi-automated quality control process including removing subjects without completed diffusion or T1-weighted scans, excessive motion, other imaging artifacts, and successful registration into a cohort-specific template space, 3284 and 3952 subjects remained for final analysis from baseline and follow-up timepoints respectively. This includes a longitudinal crossover of 2666 subjects, and a net of 4554 unique subjects. There were significantly more males than females in both baseline and follow-up samples (1741 males (53.2%) versus 1527 females (46.8%), *p* < 0.001 at baseline; 2122 males (53.7%) versus 1814 females (46.3%), *p* < 0.001 at follow-up). The age ranges at baseline were the same for the female cohort and the male cohort (107.0 to 132 months), and there was no significant difference between the average age of females (119.5 months ± 7.4 SD) and the average age of males (119.9 months ± 7.5 SD) (*p* = 0.086). While the age ranges were slightly different at the follow-up timepoint (129 to 162 months for females vs. 127 to 160 months for males), there was no significant difference between the average age of females (142.8 months ± 7.6 SD) and the average age of males (143.2 months ± 7.7 SD) (*p* = 0.080) in the follow-up sample.

### 2.2. Demographic Data

**Figure 2:**
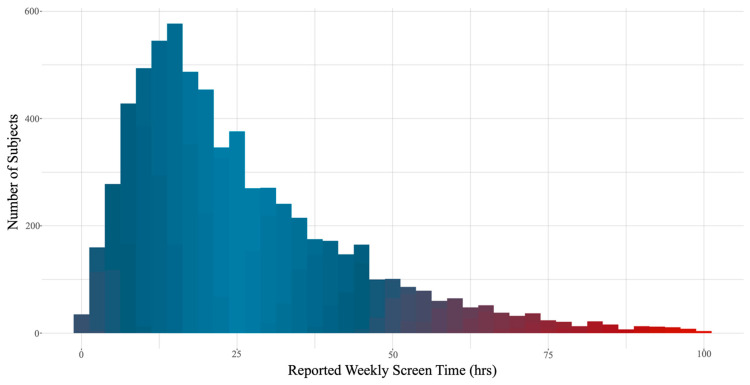
A histogram of average weekly screentime hours by subjects included in this study. Average weekly screentime hours were calculated by summation of parent responses to weekday and weekend screen time uses by activity and averaged across timepoints. The average reported weekly screen time across all subjects is 25.6 h.

Screen time data were acquired through the ABCD Youth Screen Time Survey (STQ): a parent-reported questionnaire administered at both the baseline and two-year follow-up timepoints. Respondents were asked to indicate the number of hours per day their child participated in an array of screen-based activity such as watching TV shows or movies, watching videos (on platforms such as YouTube), playing video games, texting, visiting social networking sites, and video chatting. Reported average screen usage during weekdays and on weekends across all activities were aggregated with a weighted average (accounting for five weekdays and two weekend days) to estimate the total hours per week of screen time. The screen time usage between baseline and follow-up timepoints was then averaged for each subject and used as a predictor variable for linear regression models. Exact survey questions were changed between baseline and follow-up timepoints to account for an increased granularity in screen time usage, but aggregating methods were fundamentally the same across timepoints. The change in survey methodology limits the statistical inference and longitudinal directionality possible from this analysis; however, this metric aims to establish a consistent mean weekly screentime distribution across the sample (Figure 2). Screen time was collated into a holistic measure to capture whole estimated engagement with digital devices by adolescents. While there are reasonable hypotheses as to cognitive differences between engagement in varying types of screen time, we wished to avoid introducing further potential confounders that were not adequately addressed in the study data. For example, watching videos can be performed on either a handheld device, a desktop, or a television with internet connectivity. The comment section for YouTube could possibly be engaged with in a manner similar to social media feeds or message boards. Live streams on platforms add additional social and interactive components between the user and host that are not captured in the format of the survey. This crossing of boundaries within survey categories positions the ABCD Study survey as better for capturing total engagement with digital devices and not specific forms or manners of engagement, from which additional conclusions could be drawn.

Sports involvement data, used as a proxy for physical activity, was acquired through the ABCD Longitudinal Parent Sports and Activities Involvement Questionnaire (SAIQ)—a parent-reported questionnaire administered at both the baseline and two-year follow-up timepoints. Respondents were asked to indicate the number of hours per day their child participated in a wide variety of physical activities and sports such as soccer, surfing, and horseback riding. Reported weekly hours for each activity was summed for each subject and used as a control for linear regression models.

Household income data, used as a proxy for socioeconomic status, was acquired through the ABCD Parent Demographics Survey, in which parents report total combined household income for the past 12 months. This survey was administered at both baseline and follow-up timepoints. The ABCD Study includes an oversampling of families with twins, and the final cohort included 1070 twins, 19 triplets (drawn from seven families of triplets, but in two families one subject did not pass quality control), and 563 non-twin siblings [29].

### 2.3. Imaging Data

Unprocessed T1-weighted ‘Magnetization Prepared Rapid Acquisition Gradient Echo’ (MPRAGE) MRI pulse sequences were obtained from the ABCD Study. These images were acquired with an isotropic voxel size of 1.0 × 1.0 × 1.0 mm^3^ with TE = 2.88 and TR = 2500 with a flip angle of 8 degrees and a field of view of 256 × 256 mm^2^ [23].

As described in Newman et al., 2020 [30], unprocessed dMRI images were obtained from the ABCD Study and were acquired with a multiband accelerated sequence that had an isotropic voxel size of 1.7 × 1.7 × 1.7 mm^3^ with TE = 88 ms and TR = 4100 ms. Using a multi-shell protocol, seven images were acquired at b = 0, six directions were acquired at b = 500 s/mm^2^, 15 directions were acquired at both b = 1000 s/mm^2^ and at b = 2000 s/mm^2^, and 60 directions were acquired at b = 3000 s/mm^2^ [23]. Two bidirectional field maps with reverse phase encoding were obtained at b = 0 with identical isotropic voxel sizes, TE, and TR for use in distortion correction.

Total brain volume and fractional anisotropy (FA) data were obtained from the ABCD Study baseline and follow-up releases. Volumetric metrics were calculated using an automated processing pipeline in Freesurfer version 6.0.1 [31]. FA was calculated through a diffusion tensor model using a standard, linear estimation approach with log-transformed diffusion-weighted signals, as described in Palmer et al., 2022 [32].

### 2.4. Regions of Interest

ROIs were selected based on previous association with screen time usage in functional and structural neuroimaging studies, as described by Marciano et al. 2021 [3]. Though many studies have identified a number of brain areas to be associated with digital device usage, we selected the ROIs used in this study based on strong evidence presented in this scoping review, as well as to match with ABCD Study data. A desire to include all types of screen time data, without differentiating between active and passive or by size of screen or method of interaction, drove the decision to utilize the same framework as the review. Primary clusters of activation identified by these previous studies were localized to bilateral ROIs available in the Destrieux cortical atlas [33].

### 2.5. Imaging Preprocessing and Analysis I: GMD from T1W MRI

VBM analysis was performed identically to Barrett et al., 2018 [34]. Using acquired magnetization-prepared rapid gradient-echo T1 sequence (MPRAGE), we applied voxel-based morphometry methodology [20,35]. Briefly, images were spatially normalized to standard stereotactic space through both an affine and high-dimensional nonlinear registration. MRI scans were segmented into gray and white matter and high-dimensionally fit to the Montreal Neurological Institute (MNI) standard space with the CAT12 toolbox (https://neuro-jena.github.io/cat//, accessed on 10 June 2022) in conjunction with SPM12 (https://www.fil.ion.ucl.ac.uk/spm/software/spm12/, accessed on 10 June 2022) in MATLAB, version 2022a (MathWorks, Natwick, MA, USA). To preserve the absolute volume of gray matter, segmented images were multiplied by the relative voxel volumes contained within the Jacobian determinant matrix of the deformation field. Regional GMD was measured as an average of each Destrieux atlas map within a ROI.

### 2.6. Imaging Preprocessing and Analysis II: Tissue Microstructure from dMRI

Identically to Newman et al. [25], 3T-CSD analysis was performed and was consistent with prior protocols that have been shown to result in consistent and reliable signal fraction measurements [24]. All dMRI images were corrected for thermal noise [36] and Gibbs rings [37]. “topup” and “eddy” were subsequently applied to correct for susceptibility-induced (EPI) distortions, eddy currents, and subject motion, including the Gaussian replacement of outliers [38,39,40,41]. Finally, the preprocessed image’s voxel sizes were upsampled to 1.3 × 1.3 × 1.3 mm^3^ [42,43]. Three-tissue constrained spherical deconvolution (3T-CSD) was performed on the outermost (b = 3000 s/mm^2^) [24]. Brain masks were obtained for all subjects using FSL’s Brain Extraction Tool [44].

A cohort-specific template was constructed from a sex-balanced random selection of 40 subjects’ WM-FODs at both baseline and the follow-up timepoints using symmetric diffeomorphic registration of the WM FODs [45,46]. The cohort template was then registered to stereotaxic MNI space to a b-value-matched version of the NTU-DSI-122 template to establish registration with MNI space [30]. All subjects’ maps were moved into the cohort template space, and ROIs were then warped into the common template space. Mean 3T-CSD values were then measured within each ROI. For FA, the relevant regions from the Destrieux atlas provided by the ABCD Study were averaged together to create the equivalent of the fMRI-defined ROIs by weighting each individual constituent ROI’s mean FA using that region’s volume.

### 2.7. Statistical Analysis

*Data Summarization:* Categorical variables are summarized by frequencies (*n*) and percentages (%). Continuous scaled variables are summarized by the mean, standard deviation, and range of the empirical distribution.

*GMD, FA, and Tissue Signal Fraction Regression Analysis:* Type II ANOVA models were used to predict mean ROI GMD, FA, and tissue signal fractions (i.e., extracellular isotropic, intracellular isotropic, or intracellular anisotropic) as a function of a subject’s average weekly screen time (h/week), age (years), sex (female, male), total brain volume (TBV, cm^3^), combined family income, and physical activity (h/week).

Total brain volume is known to change rapidly during the developmental period and to be associated with differential changes in WM microstructure; in a prior study, it was found that total brain volume is associated with 3T-CSD metrics [25]. Previous studies have also suggested that physical activity can positively affect the development of neuronal microstructure, particularly increases in fractional anisotropy, so we included physical activity as a covariate to disaggregate screen time effects from physical activity (or lack thereof) [47]. In all models, each child’s family and sibling relationships were accounted for as a nested random effect within site to ensure twins, triplets, and siblings were not biasing results following recommendations by Saragosa-Harris et al. [48]. All of the model predictor variables and interactions were selected a priori based on scientific merit, and were identical between each imaging-derived metric of interest, displayed as Equation (1):

Imaging Metric of Interest (GMD, FA, ECI, ICI, or ICA) ~ Screen Time + Sex + Age + Total Brain Volume + Socioeconomic Status + Physical Activity + (1|Site) + (1|Site:Family) + (1|Family:Twin) + (1|Subject).(1)

For each ROI, the concomitant variable-adjusted association between tissue signal fraction and average ST was quantified by the regression slope coefficient estimate associated with weekly ST. For each ROI, a null hypothesis test was performed to test the null hypothesis that the slope of the association between the tissue signal fraction and weekly ST is equal to 0, versus the alternative that the slope of the association between the tissue signal fraction and screen time is not equal to 0. The complete set of *p*-values from the 13 different ROIs were then subjected to the Holm–Bonferroni method for multiple comparisons to identify those ROIs, in which the ANOVA type II F-test *p*-value of the null hypothesis test was less than the adjusted *p*-value for each hypothesis. This correction was performed for each imaging metric separately.

## 3. Results

*GMD and FA Regression Analyses:* The ANOVA summaries for the mixed-effect regression models that were used to account for random nested effects from sibling relationships and random effects from the scanner site predict GMD and FA as functions of the subject’s average weekly screen time. The subject’s age, sex, household income, physical activity, and total brain volume are summarized in Table 2, which displays the number of ROIs that had a significant association with each of the predictor variables. While there are no significant relationships between weekly screen time and GMD in any of the 13 ROIs analyzed, age, sex, total brain volume, and household income were significant predictors across multiple regions. Similarly, while only the ventromedial prefrontal cortex showed significant associations between screen time and FA, age, sex, physical activity, total brain volume, and household income were significant predictors across multiple regions.

Longitudinal relationships between regional GMD and FA metrics and age were observed across all 13 ROIs. Subject age is inversely related to the GMD of every ROI, with the exception of the amygdala. Similarly, subject age is inversely related with the FA of every ROI analyzed.

*Tissue Signal Fraction Regression Analyses:* The ANOVA summaries for the mixed-effect regression models that were used to account for random nested effects from sibling relationships and random effects from the scanner site predict tissue signal fraction (i.e., extracellular isotropic CSF-like, intracellular isotropic GM-like, or intracellular anisotropic WM-like) as a function of the subject’s average weekly screen time. Subjects’ age, sex, household income, physical activity, and total brain volume are summarized in Table 3, which displays the number of ROIs that had a significant association with each of the predictor variables.

**Figure 3:**
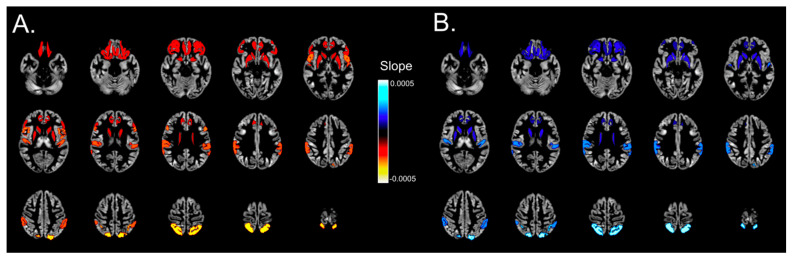
Display of significant adjusted intracellular isotropic (**A**) and intracellular anisotropic (**B**) signal fraction model slopes from analyzed ROIs. ROI maps are colored by slope (representing the change in signal fraction per hour of weekly screen usage) and displayed on the cortical ribbon of the cohort-specific template.

Significant longitudinal associations between screen time and 3T-CSD tissue microstructure were found in eight regions. A significant positive correlation was observed between screen time and ICA signal fraction in the vmPFC, the orbitofrontal cortex, the SPL, the TPJ, the caudate nucleus, and the putamen (Figure 3). In these regions and the IFG and IPL, a correspondingly significant negative correlation between screen time and ICI signal fraction was observed.

**Figure 4:**
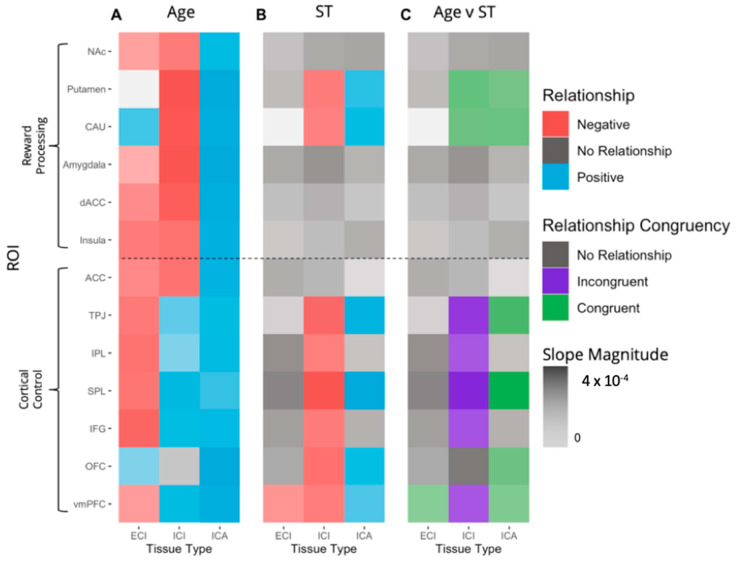
Regional relationships of both age and screen time (ST) on tissue microstructure showcasing relationship direction and strength, organized by functional associations. (**A**) Regional relationships between age and tissue microstructure characterizing typical development. All but two region/signal fraction combinations demonstrate significant relationships with age that vary in direction and strength. (**B**) Regional relationships between average weekly screen time and tissue microstructure. A total of eight out of 13 ROIs demonstrate significant relationships with screen time across at least one signal fraction. (**C**) Regional comparisons between age and screen time on microstructure. Congruency is defined as whether the relationship of age and of screen time on microstructure are in the same direction (both positive or both negative) or different directions (one positive and one negative) for a given region and tissue type.

Longitudinal relationships between 3T-CSD metrics and age are widespread, and show mixed congruency with the relationship between 3T-CSD metrics and screen time (Figure 4). Subject age is positively correlated with ICA signal across all 13 ROIs and ICI signal in the vmPFC, the IFG, the SPL, IPL, and TPJ. Age is negatively correlated with ICI signal in the ACC, insula, dACC, amygdala, CAU, putamen, and NAc. Congruency between the associations of screen time on tissue microstructure and age on tissue microstructure is defined as whether or not the slopes of these relationships share the same direction (positive or negative). Congruency was also observed in the ICA signal fraction of every significant region, as well as the ICI signal fraction in the putamen and CAU. Incongruency was observed in the ICI signal fraction of the vmPFC, IFG, SPL, IPl, and TPJ (Figure 4). Age, sex, total brain volume, and household income were also shown to significantly contribute to tissue microstructure. Full results and *p*-values from each of the covariates in each ROI are described in detail in Supplementary Table S1.

## 4. Discussion

In this longitudinal MRI study of 2666 adolescents from the ABCD Study, we have identified a relationship between parent-reported screen media usage and several measurements of brain tissue microstructure across brain regions involved in the control and reward systems. This finding highlights a complex interaction with typical age-related development that may help to explain the seemingly contradictory findings in the literature between screen time and neural development. Our observations about the effects of age on typical neural development and tissue microstructure in adolescents agrees with previous findings and paints an important contrast to our results on the effects of increased screen time. In all but two regions, we found significant relationships between subject age and tissue microstructure across tissue types. Increased subject age has a negative relationship with ECI signal fraction in every region except for the putamen (no relationship), the CAU (positive relationship), and the OFC (positive relationship). This suggests that as adolescents experience typical development, the amount of CSF/free water in these regions tends to decrease with age. Age has a consistent positive relationship with the ICA signal fraction in all ROIs, suggesting that, in these regions, the proportion of myelinated fibers increases with age. Finally, subject age has a region-dependent effect on the ICI signal fraction. In every control region analyzed—except for the OFC and ACC—subject age was positively correlated with ICI signal fraction, suggesting that adolescents develop an increased proportion of gray-matter-like tissue in these regions as they age. However, in every reward processing region analyzed, subject age was negatively correlated with ICI signal fraction. This subcortical response to typical development more closely matches previous findings with less specific metrics, though the ICI signal fraction does not necessarily correlate with GM volume.

The differential effect of aging on white matter microstructure in control and reward regions is further supported by recent research conducted on ABCD data. The effects of age on metrics derived from restriction spectrum imaging (RSI), a diffusion-weighted imaging-derived model that decomposes signal from a voxel into hindered, restricted, and free compartments—analogous to ICI, ICA, and ECI respectively—were shown to agree broadly with the presented findings [32]. Palmer et al. report that within WM, voxel-wise relative measurements of directional diffusion (RDF) decreased with age compared to isotropic diffusion, an identical finding to previously published 3T-CSD findings in the ABCD cohort [25]. Additionally, Palmer et al. report identical directions for hindered and restricted model compartments analogous to the 3T-CSD model’s ICI and ICA compartments in the subcortical GM regions (hindered and ICI decrease with age, whereas restricted and ICA increase). Model differences may account for the difference in slope observed between the two studies. RND, composed of the second- and fourth-order spherical harmonic coefficients, may best represent the two largest fiber bundles within a particular voxel, whereas in the 3T-CSD model used in this paper, the ICA signal fraction comprises all detectable WM-like signals from up to four fiber populations, meaning that ICA may be more sensitive to smaller fibers. The stronger effect of age on ICA signal fraction observed in this study may be due to the greater development of these smaller diameter fibers. By relying entirely on relative metrics comparing signal from compartments, rather than absolute signal change, 3T-CSD may potentially be more sensitive to small-scale changes in brain microstructure than RSI.

In contrast to the effects of aging, increased screen time was associated with a complex series of changes in neural tissue signal fractions in eight of the ROIs, including six regions involved in the control system and two involved in reward processing. The only observed association between screen time and ECI was found in the vmPFC, where increased media use had a negative relationship with CSF-like signal fraction. Similar to the association between age and ICA signal fraction, a positive correlation was the only significant relationship observed between screen time and ICA (six regions). The putamen, amygdala, TPJ, SPL, OFC, and vmPFC were congruent in the associations between screen time and age on ICA signal fraction. These results suggest that increased screen time accelerates the positive trend of ICA development in these regions beyond age-related increases. Significant negative relationships between screen time and ICI signal fraction were observed in eight of the ROIs. However, these associations were incongruent with age-related development within cortical control regions (TPJ, IPL, SPL, IFG, OFC, and vmPFC) but congruent with subcortical reward processing systems. Thus, these results suggest that screen time has a consistent relationship with microstructure in significant ROIs, accelerating ICA development and decelerating ICI development. However, considering the apparent differences in developmental trajectories between control and reward regions, these results may indicate a fundamental difference in how screen time affects regions involved with the control and reward systems, accelerating development in reward regions and demonstrating mixed responses between signal fractions in control regions.

The contrast between the effects of screen time on tissue microstructure and the effects of age on tissue microstructure characterizes an important relationship between increased screen time and neural development on a cellular level. The relative increase in ICA signal fraction in all significant regions suggests that screen time is associated with a more rapid maturation of axonal innervation in the cortex in these regions. The universal decrease in ICI signal fraction across significant regions is vaguer, but is potentially the result of a decrease in the net number of cells in these regions. The slope of both these relationships was highly similar in magnitude (10^−3^ to 10^−2^). While not a true statistical comparison and using slightly different ranges (0–100 h for screen time vs. 107–132 months at baseline for age), this does suggest that screen time had powerful effects on neuronal microstructure similar to typical aging. These results, taken together, suggest that in response to screen time, significantly affected regions of the brain demonstrate a decreasing neural population, increasing axonal myelination, or both simultaneously, indicating that the wiring of the adolescent brain might be maturing more rapidly, possibly at the consequence of the typical neurogenesis observed in the cortex in this age range. However, the cellular responses to increased screen time that underly the relationships observed in this study are relatively ambiguous, as the signal fraction analysis used in this study does not pin changes in tissue microstructure to specific cell types or changes, particularly for ICI signal fraction. Similarly, because this model focuses on the relative presence of signal type, whether or not there are effects on the absolute amount of tissue contributing to each signal type cannot be determined.

One possible explanation for the lack of significant FA results in the face of a changing microstructural environment is the lack of orientation resolution across white matter fibers in this metric. By only modeling unidirectional white matter microstructure in each voxel, the FA model is likely not as sensitive as the 3T-CSD model for this analysis, particularly because of the complex white matter directionality in the cortex. However, 3T-CSD is able to measure the total signal in a directionless manner. The lack of significant longitudinal effects observed between increased screen time and both GMD and FA, but the presence of such results in 3T-CSD metrics, highlights both the sensitivity of this technique and importance of multi-dimensional voxel analysis.

While this study found no significant effect of screen time on GMD cross-sectionally, our hypothesis of longitudinal effects, based primarily on the findings of Takeuchi et al. [12], was not supported by the absence of significant results. Several key differences in the structure of the present study may explain this discrepancy. While the subject age is comparable between studies, the number of subjects and amount of time between MRI visits was different between studies (223 subjects in Takeuchi et al. vs. 2666 subjects here, 3 years between scans in Takeuchi et al. vs. 2 years between scans here). Critically, there is also a difference between explanatory variables: Takeuchi et al. observed the effects of internet usage, and the present study observed the effects of a variety of screen-based activities. While consistent with the current literature, our longitudinal FA results also opposed our hypothesis, only revealing significant relationships between screen time and WM structure in the vmPFC.

This study also faces a number of limitations, including the usage of a weighted mean of screen time between timepoints instead of genuinely longitudinal changes and the exclusive reliance on Siemens scanner data. Though the image processing limitations were intentionally chosen to enhance the reliability and accuracy of the results, it is possible that the results of this study are less broadly generalizable to other scanner manufacturers or other image acquisition schemes. A more rigorously controlled study may uncover additional relationships between neuronal microstructure and adolescent screen time usage. There are further confounding variables that were not included in this study that may have affected results, such as pubertal status, sleep duration/quality, BMI, mental health symptoms, and other relevant factors. Further investigation into the relationship between screen time and neuronal development should consider these relationships, although our previous work has suggested that puberty primarily drives an intracellular isotropic, instead of an intracellular anisotropic, signal in this same cohort [25] and some of these factors such as BMI and sleep duration/quality are known to be associated with socioeconomic status [49,50]. The use of physical activity in this study was designed to be as comprehensive as possible given the use of the pre-existing ABCD Study data. A wide variety of activities were included in the physical activity data, including unorganized sport and individual activities. The wording of the physical activity questionnaire specified the maximum amount of activity during the most intensive week across the entire year [51], and the provided responses suggested that for more intense organized sport, many parents reported participation in excess of full time hours at school (i.e., >40 h/week), likely indicating attendance at camps or tournaments for organized activity. While intensity of activity was not directly measured, we believe this method of phrasing and aggregating physical activity was perhaps the closest possible incorporation of intensity of activity into the physical activity metric. Data for each participant was also collected using a FitBit accelerometer worn over the course of 1 week; however, we determined that this was too narrow of a window to provide meaningful generalizability over the broader 2-year period of the study. While ultimately the physical activity metric used as a control in this study remains imperfect and limited, we believe it does attempt to represent physical intensity vs. sedentary behavior and that non-sport physical activities are included. It should also be considered a limitation of this study that screen time was reported by parents, which could be vulnerable to recall errors and estimation biases. Future work could incorporate objective measures obtained through digital records of device usage, which would also enable more specific data tracking.

There are a host of potential factors influencing the observed relationship between screen time and tissue microstructure, and it is highly likely that the effects of screen time on cellular microstructure in adolescents are related to the contents of screen usage rather than the medium itself. Possible components that may be driving these changes in cognitive and reward pathways are the over-stimulating and easily gratifying nature of most screen-based activities, which are causing rapid maturation of the reward pathway while inhibiting traditional cognitive development. This explanation supports the negative behavioral and cognitive outcomes that have previously been associated with increased screen usage. While this is an observational study, the clinical implications suggest that alterations in age-related developmental trajectories, revealing themselves as early or late patterns of maturation, may be more indicative of heavy screen time usage than de novo changes or alterations of specific circuits. This may additionally provide important background for future studies exploring cessation or reduction in screen time strategies. By tying the effects of screen time to age-related neural maturation, it may potentially be useful to describe changes as accelerating or decelerating neuronal changes. Future studies should rigorously control or record factors like screen size, degree of participant engagement, ‘gamification’ of the system in question, and social components to better associate neuronal changes with specific behaviors. In sum, by showing the acceleration and deceleration of typical development, these results suggest screen time in adolescents either over- or under-stimulates specific cognitive processing networks, with unknown long-term consequences.

In summary, though this study is observational in design, our quantifications of the effects of screen time on neural development in adolescents suggest cellular changes in the brain might be the consequences of habitual media usage. Further research is required to distinguish the specific effects of different screen uses on the adolescent brain, and more rigorously controlled studies manipulating screen time will be necessary to establish causality.

## Figures and Tables

**Table 1:** Regions of interest selected for this study based on previous functional or structural associations with screen media consumption. Region indices refer to the labels used in Figure 1. Regions are divided based on their roles in either the control or reward systems [3].

Figure 1 Index	Region	Associated System
1	vmPFC	Control
2	OFC	Control
3	IFG	Control
4	SPL	Control
5	IPL	Control
6	TPJ	Control
7	ACC	Control
8	Insula	Reward
9	dACC	Reward
10	Amygdala	Reward
11	CAU	Reward
12	Putamen	Reward
13	NAc	Reward

**Table 2:** Number of ROIs (out of a total of 13) where each associated variable remained significantly associated with GMD and FA metrics after implementation of the Holm–Bonferroni method for multiple comparisons (*p* < 0.05).

Metric	Screen Time	Age	Sex	TBV	Household Income	Physical Activity
GMD	0 (0)	13 (100)	10 (76.9)	13 (100)	6 (46.2)	0 (0)
FA	1 (7.7)	13 (100)	9 (69.2)	8 (61.5)	4 (30.8)	0 (0)

**Table 3:** Number of ROIs (out of a total of 13) where each associated variable remained significantly associated with each tissue signal fraction compartment after implementation of the Holm–Bonferroni method for multiple comparisons (*p* < 0.05).

Signal Fraction	Screen Time Number (%)	Age Number (%)	Sex Number (%)	TBV Number (%)	Family Income Number (%)	Physical Activity Number (%)
ECI	1 (7.7)	12 (92.3)	4 (30.8)	5 (38.5)	7 (53.8)	3 (23.1)
ICI	8 (61.5)	12 (92.3)	1 (7.7)	8 (61.5)	7 (53.8)	0 (0)
ICA	6 (46.2)	13 (100)	3 (23.1)	4 (30.8)	4 (30.8)	0 (0)

## Data Availability

Data from this study is freely available from the National Institutes of Health NDAR database, and at https://abcdstudy.org/.

## Supplementary Materials

The following supporting information can be downloaded at: https://www.mdpi.com/article/10.3390/children13070969/s1. Table S1: Full statistical results and *p*-values from each of the covariates in each ROI from the primary 3T-CSD analysis.

## References

[1] Pandya A., Lodha P. (2021). Social Connectedness, Excessive Screen Time During COVID-19 and Mental Health: A Review of Current Evidence. Front. Hum. Dyn..

[2] Campbell J.S., Leppert I.R., Narayanan S., Boudreau M., Duval T., Cohen-Adad J., Pike G.B., Stikov N. (2018). Promise and Pitfalls of G-Ratio Estimation with MRI. Neuroimage.

[3] Marciano L., Camerini A.-L., Morese R. (2021). The Developing Brain in the Digital Era: A Scoping Review of Structural and Functional Correlates of Screen Time in Adolescence. Front. Psychol..

[4] Paus T. (2005). Mapping Brain Maturation and Cognitive Development during Adolescence. Trends Cogn. Sci..

[5] Sowell E.R., Peterson B.S., Thompson P.M., Welcome S.E., Henkenius A.L., Toga A.W. (2003). Mapping Cortical Change across the Human Life Span. Nat. Neurosci..

[6] Gogtay N., Thompson P.M. (2010). Mapping Gray Matter Development: Implications for Typical Development and Vulnerability to Psychopathology. Brain Cogn..

[7] Lissak G. (2018). Adverse Physiological and Psychological Effects of Screen Time on Children and Adolescents: Literature Review and Case Study. Environ. Res..

[8] Luna B., Paulsen D.J., Padmanabhan A., Geier C. (2013). The Teenage Brain: Cognitive Control and Motivation. Curr. Dir. Psychol. Sci..

[9] Schweizer S., Gotlib I.H., Blakemore S.-J. (2020). The Role of Affective Control in Emotion Regulation during Adolescence. Emotion.

[10] Nikkelen S.W.C., Valkenburg P.M., Huizinga M., Bushman B.J. (2014). Media Use and ADHD-Related Behaviors in Children and Adolescents: A Meta-Analysis. Dev. Psychol..

[11] Maras D., Flament M.F., Murray M., Buchholz A., Henderson K.A., Obeid N., Goldfield G.S. (2015). Screen Time Is Associated with Depression and Anxiety in Canadian Youth. Prev. Med..

[12] Takeuchi H., Taki Y., Asano K., Asano M., Sassa Y., Yokota S., Kotozaki Y., Nouchi R., Kawashima R. (2018). Impact of Frequency of Internet Use on Development of Brain Structures and Verbal Intelligence: Longitudinal Analyses. Hum. Brain Mapp..

[13] Rodriguez-Ayllon M., Derks I.P.M., Van Den Dries M.A., Esteban-Cornejo I., Labrecque J.A., Yang-Huang J., Raat H., Vernooij M.W., White T., Ortega F.B. (2020). Associations of Physical Activity and Screen Time with White Matter Microstructure in Children from the General Population. NeuroImage.

[14] Horowitz-Kraus T., Hutton J.S. (2018). Brain Connectivity in Children Is Increased by the Time They Spend Reading Books and Decreased by the Length of Exposure to Screen-Based Media. Acta Paediatr..

[15] Sherman L.E., Greenfield P.M., Hernandez L.M., Dapretto M. (2018). Peer Influence Via Instagram: Effects on Brain and Behavior in Adolescence and Young Adulthood. Child. Dev..

[16] Sherman L.E., Hernandez L.M., Greenfield P.M., Dapretto M. (2018). What the Brain ‘Likes’: Neural Correlates of Providing Feedback on Social Media. Soc. Cogn. Affect. Neurosci..

[17] Efraim M., Kirwan C.B., Muncy N.M., Tucker L.A., Kwon S., Bailey B.W. (2021). Acute After-School Screen Time in Children Decreases Impulse Control and Activation toward High-Calorie Food Stimuli in Brain Regions Related to Reward and Attention. Brain Imaging Behav..

[18] Wang H., Zhou X., Lu C., Wu J., Deng X., Hong L. (2011). Problematic Internet Use in High School Students in Guangdong Province, China. PLoS ONE.

[19] Hong S.-B., Kim J.-W., Choi E.-J., Kim H.-H., Suh J.-E., Kim C.-D., Klauser P., Whittle S., Yűcel M., Pantelis C. (2013). Reduced Orbitofrontal Cortical Thickness in Male Adolescents with Internet Addiction. Behav. Brain Funct..

[20] Mechelli A., Price C., Friston K., Ashburner J. (2005). Voxel-Based Morphometry of the Human Brain: Methods and Applications. Curr. Med. Imaging Rev..

[21] Dhollander T., Connelly A. A Novel Iterative Approach to Reap the Benefits of Multi-Tissue CSD from Just Single-Shell (+B= 0) Diffusion MRI Data. Proceedings of the International Society of Magnetic Resonance in Medicine 2016.

[22] Jeurissen B., Tournier J.-D., Dhollander T., Connelly A., Sijbers J. (2014). Multi-Tissue Constrained Spherical Deconvolution for Improved Analysis of Multi-Shell Diffusion MRI Data. NeuroImage.

[23] Casey B.J., Cannonier T., Conley M.I., Cohen A.O., Barch D.M., Heitzeg M.M., Soules M.E., Teslovich T., Dellarco D.V., Garavan H. (2018). The Adolescent Brain Cognitive Development (ABCD) Study: Imaging Acquisition across 21 Sites. Dev. Cogn. Neurosci..

[24] Newman B.T., Dhollander T., Reynier K.A., Panzer M.B., Druzgal T.J. (2020). Test–Retest Reliability and Long-term Stability of Three-tissue Constrained Spherical Deconvolution Methods for Analyzing Diffusion MRI Data. Magn. Reson. Med..

[25] Newman B.T., Patrie J.T., Druzgal T.J. (2023). An Intracellular Isotropic Diffusion Signal Is Positively Associated with Pubertal Development in White Matter. Dev. Cogn. Neurosci..

[26] Newman B.T., Dhollander T., Druzgal T.J. Investigating the Effect of Diffusion MRI Acquisition Parameters on Free Water Signal Fraction Estimates from 3-Tissue CSD Techniques. Proceedings of the 28th ISMRM Annual Meeting.

[27] Pannek K., Guzzetta A., Colditz P.B., Rose S.E. (2012). Diffusion MRI of the Neonate Brain: Acquisition, Processing and Analysis Techniques. Pediatr. Radiol..

[28] Genc S., Tax C.M., Raven E.P., Chamberland M., Parker G.D., Jones D.K. (2020). Impact of B-value on Estimates of Apparent Fibre Density. Hum. Brain Mapp..

[29] Iacono W.G., Heath A.C., Hewitt J.K., Neale M.C., Banich M.T., Luciana M.M., Madden P.A., Barch D.M., Bjork J.M. (2018). The Utility of Twins in Developmental Cognitive Neuroscience Research: How Twins Strengthen the ABCD Research Design. Dev. Cogn. Neurosci..

[30] Newman B., Untaroiu A., Druzgal T. A Novel Diffusion Registration Method with the NTU-DSI-122 Template to Transform Free Water Signal Fraction Maps to Stereotaxic Space. Proceedings of the 28th ISMRM Annual Meeting.

[31] Fischl B. (2012). FreeSurfer. Neuroimage.

[32] Palmer C.E., Pecheva D., Iversen J.R., Hagler D.J., Sugrue L., Nedelec P., Fan C.C., Thompson W.K., Jernigan T.L., Dale A.M. (2022). Microstructural Development from 9 to 14 Years: Evidence from the ABCD Study. Dev. Cogn. Neurosci..

[33] Destrieux C., Fischl B., Dale A., Halgren E. (2010). Automatic Parcellation of Human Cortical Gyri and Sulci Using Standard Anatomical Nomenclature. Neuroimage.

[34] Barrett M.J., Blair J.C., Sperling S.A., Smolkin M.E., Druzgal T.J. (2018). Baseline Symptoms and Basal Forebrain Volume Predict Future Psychosis in Early Parkinson Disease. Neurology.

[35] Ashburner J., Friston K.J. (2000). Voxel-Based Morphometry—The Methods. NeuroImage.

[36] Tournier J.-D., Smith R., Raffelt D., Tabbara R., Dhollander T., Pietsch M., Christiaens D., Jeurissen B., Yeh C.-H., Connelly A. (2019). MRtrix3: A Fast, Flexible and Open Software Framework for Medical Image Processing and Visualisation. NeuroImage.

[37] Kellner E., Dhital B., Kiselev V.G., Reisert M. (2016). Gibbs-ringing Artifact Removal Based on Local Subvoxel-shifts. Magn. Reson. Med..

[38] Andersson J.L., Skare S., Ashburner J. (2003). How to Correct Susceptibility Distortions in Spin-Echo Echo-Planar Images: Application to Diffusion Tensor Imaging. Neuroimage.

[39] Andersson J.L., Graham M.S., Zsoldos E., Sotiropoulos S.N. (2016). Incorporating Outlier Detection and Replacement into a Non-Parametric Framework for Movement and Distortion Correction of Diffusion MR Images. Neuroimage.

[40] Andersson J.L., Sotiropoulos S.N. (2016). An Integrated Approach to Correction for Off-Resonance Effects and Subject Movement in Diffusion MR Imaging. Neuroimage.

[41] Smith S.M., Jenkinson M., Woolrich M.W., Beckmann C.F., Behrens T.E., Johansen-Berg H., Bannister P.R., De Luca M., Drobnjak I., Flitney D.E. (2004). Advances in Functional and Structural MR Image Analysis and Implementation as FSL. Neuroimage.

[42] Greenspan H. (2009). Super-Resolution in Medical Imaging. Comput. J..

[43] Sotiropoulos S.N., Jbabdi S., Xu J., Andersson J.L., Moeller S., Auerbach E.J., Glasser M.F., Hernandez M., Sapiro G., Jenkinson M. (2013). Advances in Diffusion MRI Acquisition and Processing in the Human Connectome Project. Neuroimage.

[44] Smith S.M. (2002). Fast Robust Automated Brain Extraction. Hum. Brain Mapp..

[45] Raffelt D., Tournier J.-D., Fripp J., Crozier S., Connelly A., Salvado O. (2011). Symmetric Diffeomorphic Registration of Fibre Orientation Distributions. NeuroImage.

[46] Raffelt D., Tournier J.-D., Crozier S., Connelly A., Salvado O. (2012). Reorientation of Fiber Orientation Distributions Using Apodized Point Spread Functions. Magn. Reson. Med..

[47] Chaddock-Heyman L., Erickson K.I., Kienzler C., Drollette E.S., Raine L.B., Kao S.-C., Bensken J., Weisshappel R., Castelli D.M., Hillman C.H. (2018). Physical Activity Increases White Matter Microstructure in Children. Front. Neurosci..

[48] Saragosa-Harris N.M., Chaku N., MacSweeney N., Williamson V.G., Scheuplein M., Feola B., Cardenas-Iniguez C., Demir-Lira E., McNeilly E.A., Huffman L.G. (2022). A Practical Guide for Researchers and Reviewers Using the ABCD Study and Other Large Longitudinal Datasets. Dev. Cogn. Neurosci..

[49] McLaren L. (2007). Socioeconomic Status and Obesity. Epidemiol. Rev..

[50] Moore P.J., Adler N.E., Williams D.R., Jackson J.S. (2002). Socioeconomic Status and Health: The Role of Sleep. Biopsychosoc. Sci. Med..

[51] Barch D.M., Albaugh M.D., Avenevoli S., Chang L., Clark D.B., Glantz M.D., Hudziak J.J., Jernigan T.L., Tapert S.F., Yurgelun-Todd D. (2018). Demographic, Physical and Mental Health Assessments in the Adolescent Brain and Cognitive Development Study: Rationale and Description. Dev. Cogn. Neurosci..

